# Widespread distribution of lymphatic vessels in human dura mater remote from sinus veins

**DOI:** 10.3389/fcell.2023.1228344

**Published:** 2023-09-19

**Authors:** César Luis Vera Quesada, Shreyas Balachandra Rao, Reidun Torp, Per Kristian Eide

**Affiliations:** ^1^ Department of Neurosurgery, Oslo University Hospital-Rikshospitalet, Oslo, Norway; ^2^ Institute of Clinical Medicine, Faculty of Medicine, University of Oslo, Oslo, Norway; ^3^ Division of Anatomy, Department of Molecular Medicine, Institute of Basic Medical Sciences, University of Oslo, Oslo, Norway

**Keywords:** cerebral meninges, human dura mater, meningeal lymphatic vessels, dural sinus veins, cerebrospinal fluid, neuro-immunology

## Abstract

**Background and purpose:** Previous experimental studies have shown that meningeal lymphatic vessels are located primarily along the walls of the dural sinus veins. Whether they are more widespread throughout human dura mater has presently not been characterized. The present study explored in humans whether meningeal lymphatic vessels may be identified remote from the sinus veins and whether they differ in the various location of dura mater.

**Methods:** We included 15 patients who underwent neurosurgery, in whom dura mater was removed as part of the planned procedure. Tissue was prepared for immunohistochemistry using the lymphatic endothelial cell markers lymphatic vessel endothelial hyaluronan receptor 1 protein (LYVE-1), podoplanin and vascular endothelial growth factor receptor 3 (VEGFR3).

**Results:** Lymphatic endothelial cell positive cells were found in dura mater at the posterior fossa (n = 8), temporal skull base (n = 5), frontal convexity (n = 1), and cranio-cervical junction (n = 1). They were most commonly seen remote from blood vessels, but also occurred along blood vessels, and seemed to be most abundant at the skull base.

**Conclusion:** The present observations show that human lymphatic vessels are widespread in dura mater, not solely lining the dural sinuses.

## 1 Introduction

It has been known for centuries that the dura mater may harbor lymphatic vessels (Sandrone et al., 2019), but the role of meningeal lymphatic vessels (MLVs) regained renewed interest since 2015 (Louveau et al., 2015; Aspelund et al., 2015), when functional MLVs were first reported. These MLVs were found to carry substances and waste from the cerebrospinal fluid (CSF), and impairing their function reduced efflux of toxic waste substances from CSF (Zou et al., 2019; Da Mesquita et al., 2021; Ding et al., 2021). Previous studies about MLVs primarily focused on their location along the dural sinus vein walls (Louveau et al., 2015; Visanji et al., 2018), though some data suggested a role of MLVs at skull base for CSF efflux (Ma et al., 2017; Jacob et al., 2022). While it has been attempted to estimate distribution of MLVs in human dura utilizing magnetic resonance imaging (Jacob et al., 2022), the anatomical distribution of MLVs in human dura mater utilizing immunohistochemistry has been less characterized. We recently reported existence of MLVs lateral to the superior sagittal sinus in the right frontal region of patients (Vera Quesada et al., 2023).

The present study asked whether MLVs in patients are distributed throughout the dura mater and not confined solely to the walls of the dural sinus veins. We therefore examined whether MLVs were identifiable in human dura mater remote from dural sinus veins, and whether MLVs in different locations show variable dimensions.

## 2 Materials and methods

### 2.1 Ethical approvals

The study was approved by the Regional Committee for Medical and Health Research Ethics (REK) of Health Region South-East, Norway (Approval number 12639 and 28882) and The Institutional Review Board of Oslo university hospital (2012/14287 and 2011/19311) approved the study. Oral and written informed consent was obtained prior to tissue sampling.

### 2.2 Experimental design

The study design was prospective and observational.

### 2.3 Participants

Study participants were neurosurgical patients undergoing surgery in whom removal of dura mater was part of the required surgery.

### 2.4 Tissue sampling, preparation and immunohistochemistry

Dura mater samples were obtained during neurosurgery for treatment of meningioma, middle cerebral artery (MCA) aneurysm, cavernoma or pineal cyst. These samples consisted of healthy dura mater (non-tumoral) and measured between four to 9 mm in diameter depending on each individual surgical approach and about 1 mm thick. As previously described (Vera Quesada et al., 2023), the samples were immediately frozen on dry ice in Optimal Cutting Temperature (OCT) medium (Richard-Allan Scientific™ Neg-50™, Thermo Fisher Scientific; Cat#6502) and stored at −80°C until sectioning in a Cryostat NX70 from Thermo Scientific with vacutome. Since it was hard to determine the rostro-caudal or medio-lateral orientation in the horizontal plane of the tissue, the sections were either sagittal or coronal and about 30 μm thick. The cranial (outer) vs. arachnoid (inner) orientation in the vertical plane of the tissue was deciphered through microscopy. Sections were immediately mounted on Superfrost™ Plus microscopy slides (Thermo Fisher Scientific) and stored at −80°C until immunohistochemistry (IHC). Prior to IHC, the slides were thawed to room temperature for about 5 min before the sections were fixed on the slide with 0.5% para-formaldehyde (PFA) for 10 min.

For immunolabeling with lymphatic vessel endothelial hyaluronan receptor 1 protein (LYVE-1), vascular endothelial growth factor receptor 3 (VEGFR3) or podoplanin (PDPN), the sections were rinsed immediately after fixation three times for 5 min each in 0.01 M phosphate buffer saline (PBS). The sections were blocked for 60 min with a blocking solution (10% normal donkey serum, 1% bovine serum albumin (BSA), 0.5% Triton X-100 in PBS) to reduce unspecific binding of antibodies. Subsequently, they were incubated in blocking solution containing 0.01% sodium acid with the primary antibodies against LYVE-1 (polyclonal rabbit anti-LYVE-1: Abcam cat#ab33682, RRID:AB_881387, 1:500 dilution), VEGFR3 (polyclonal rabbit anti-VEGFR3: Abcam cat#ab27278, RRID:AB_470949.1:250 dilution) or PDPN (monoclonal mouse anti-PDPN: GeneTex cat#50043, RRID:AB_11166297, 1:1,000 dilution) overnight at 4°C. The next day, the sections were rinsed 3 times for 10 min each in 0.01 M PBS following incubation in the blocking solution with the secondary antibody (Cy3 donkey anti-rabbit; Jackson ImmunoResearch Labs; Cat#:711-165-152; RRID:AB_2307443, 1:250 dilution or Cy2 donkey anti-mouse; Jackson ImmunoResearch Labs; Cat#:715-225-150; RRID:AB_2340826) for 30 min and another 30 min with the secondary antibody plus DyLight^®^ 649 conjugated tomato lectin (LEL, TL; Vector labs; Cat#: DL-1178) to stain blood vessels. Thereafter, the sections were rinsed again 3 times for 10 min each in 0.01 M PBS. Afterwards, Hoechst 33258 was used for nuclear staining (Thermo Fisher Scientific; Cat#: H3569; RRID:AB_2651133, 1:5,000 dilution) for 5 min. Finally, the sections were rinsed in distilled water, twice for 5 min, and cover-slipped using ProLong™ Gold Antifade Mountant (Thermo Fisher Scientific; Cat#: P36934; RRID:SCR_015961).

As positive controls, we added mouse liver and spleen sections to validate all 3 antibodies (Supplementary Figure S1). As for negative controls, some consecutive dura sections were incubated without the primary anti-LYVE-1/PDPN/VEGFR3 antibodies (Supplementary Figure S2).

On average, 12 sections were analyzed per sample. For microscopy and measurements, an LSM 710 confocal microscope (Carl Zeiss Microscopy) at magnification of 20x or 63x w/oil immersion objective was used to acquire high-resolution images. Tissue specimens were processed using the Zen Blue software (Carl Zeiss Microscopy). Quantitative measurements of lymphatic vessels were done using the Zen Blue software, utilizing the sections where most surface area was visually labelled. Linear measures of lumen, wall thickness and total width were made.

The cranial and arachnoid sides of the dura mater were distinguished by the overall collagen appearance and cellular density. Collagen fibers are oriented more uniformly with a low density of fibroblasts and cells towards the cranial side of the dura while the arachnoid side exhibits less organized collagen fibers due to the high density of cells and vessel structures (Frederickson, 1991; Haines et al., 1993; Protasoni et al., 2011).

### 2.5 Statistical analysis

Statistical analyses utilized SPSS version 27 (IBM Corporation, Armonk, NY, USA). We determined differences between groups using analysis of variance (ANOVA) with *post hoc* Bonferroni corrections for continuous variables and the Chi-square test for categorical variables. Two-tailed *p*-values of less than 5% were accepted as statistically significant.

## 3 Results

### 3.1 Study cohort

**FIGURE 1:**
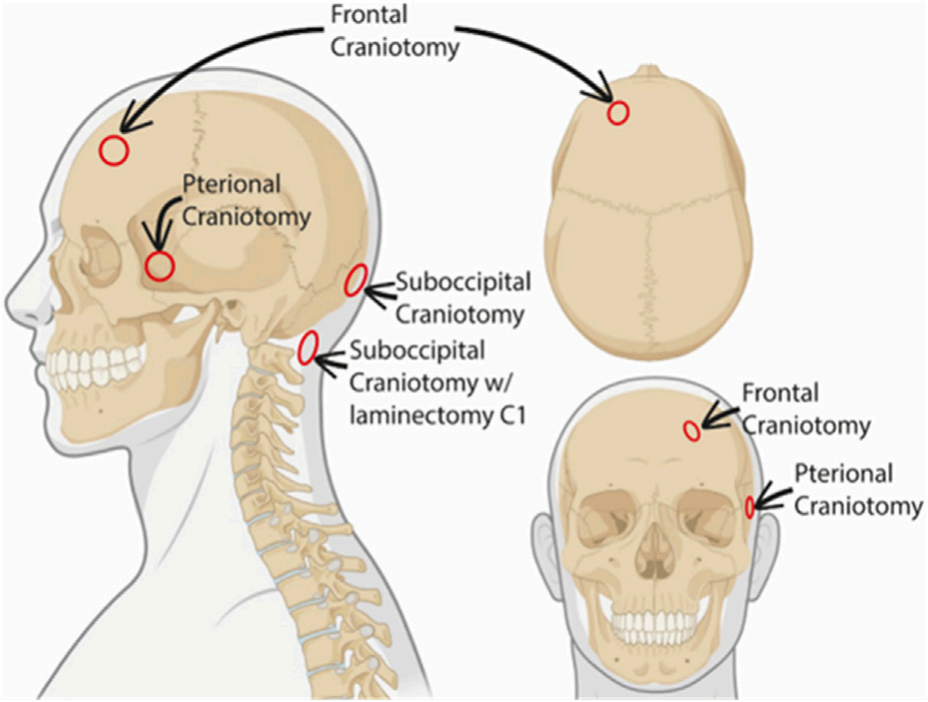
Schematic representation of dura biopsy locations. The different sites where the dural biopsies were obtained are indicated in the schematic illustration. Created with BioRender.com.

The study included fifteen individuals who underwent neurosurgical treatments where dura mater samples were obtained during the following neurosurgical procedures (Figure 1).

- Suboccipital craniotomy where dura was obtained from the lower occipital midline (n = 8).
- Pterional craniotomy where dura was obtained from the temporal dura at the level of the sphenoid wing (n = 4).
- Pretemporal craniotomy where dura was obtained from the temporal skull base (n = 1).
- Frontal craniotomy where dura was obtained from the frontal convexity >3-4 cm lateral to the superior sagittal sinus (n = 1).
- Suboccipital craniotomy with laminectomy posterior C1 where dura was obtained at the posterior C1 level (n = 1).

The patients did not differ significantly in terms of age or gender and there were no adverse events during the extraction of the dura biopsy. More detailed demographic information of these individuals is provided in Table 1.

**TABLE 1:** Patient material.

	Total	Location of dura samples			
		Posterior fossa	Temporal scull base	Frontal convexity	Cranio-cervical junction
*Demographic*					
N	15	8	5	1	1
Age (yrs)	54.2 ± 13.7	51.5 ± 14.2	61.2 ± 14.3	47.0	48.0
Sex (f/m)	8/7	4/4	2/3	1/0	1/0
***Surgical approach*** (***dura location***)					
Suboccipital (dura lower midline)	8/15 (53.3%)	8/8 (100%)	-	-	-
Pterional (caudal temporal fossa)	4/15 (26.7%)	-	4/5 (80%)	-	-
Pretemporal (temporal skull base)	1/15 (6.7%)	-	1/5 (20%)	-	-
Frontal (frontal convexity)	1/15 (6.7%)	-	-	1/1 (100%)	-
Craniocervical (C1)	1/15 (6.7%)	-	-	-	1/1 (100%)

In all 15 subjects, lymphatic vessels were found labeled with either LYVE-1, VEGFR3 and/or PDPN.

### 3.2 Lymphatic vessels in dura mater of posterior fossa

**FIGURE 2:**
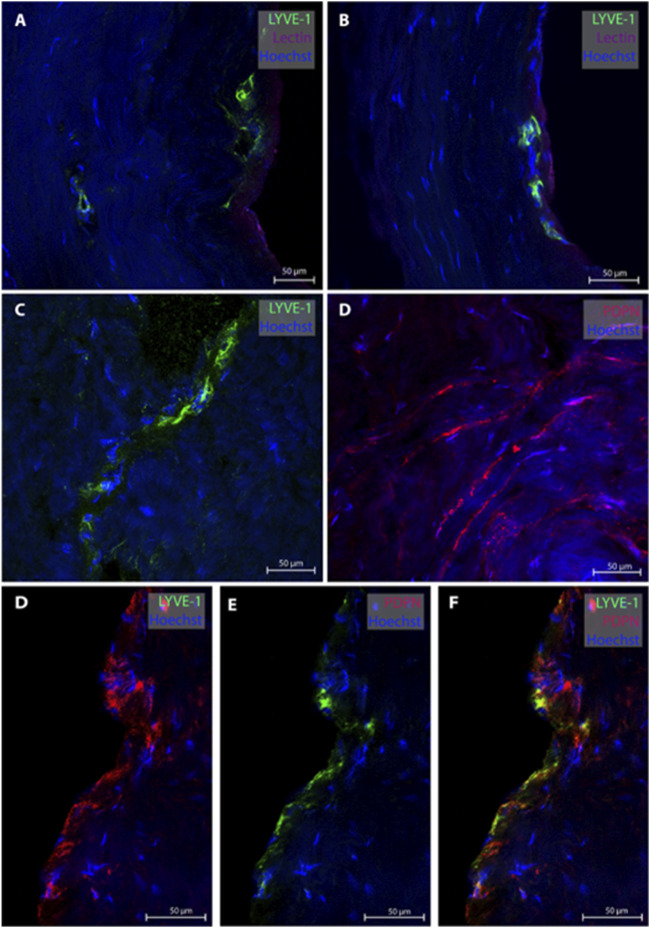
Lymphatic vessels in dura mater of posterior fossa. Representative images of lymphatic vessels in the posterior fossa. Panels **(A, B)** are sections from patient #1, panel **(C)** from patient #14 and panel D from patient#12. Panels **(D–F)** are a lymphatic vessel co-labeled with LYVE-1 and PDPN from patient #7. Negative controls of panels D and F can be found in Supplementary Figures S2D, F.

From the posterior fossa, dura mater samples from eight subjects (patients #1, 5, 6, 7, 8, 9, 12, 13 and 14) were obtained, and 20 MLVs were examined (Table 2). Representative images are shown in Figure 2. In this region, lymphatic vessel in the vicinity of blood vessels were seen in only one individual. The MLVs in the posterior fossa had an average lumen of 11.3 ± 7.7 µm, a wall thickness of 3.4 ± 2.3 µm and a total width of 20.5 ± 13.8 µm (Table 2). MLVs in a cluster-like structure with LYVE-1 immunoreactive cells was obtained in only one sample.

**TABLE 2:** Characteristics of lymphatic structures in different locations.

	Total	Location of dura samples			
		Posterior fossa	Temporal scull base	Frontal convexity	Cranio-cervical junction
***N***	15	8	5	1	1
***Number of patients presenting with***					
Lymphatic vessels with blood vessels (n; %)	4/15 (26.7%)	1/8 (13%)	3/5 (60%)	0	0
Lymphatic vessels without blood vessels (n; %)	15/15 (100%)	8/8 (100%)	5/5 (100%)	1/1 (100%)	1/1 (100%)
Clusters of LYVE-1-expressing cells (n; %)	1/15 (6.7%)	1/8 (13%)	0	0	0
***Quantitative characteristics of lymphatic vessels***					
Number of examined lymphatic vessels	60	20	26	3	11
Lumen diameter (µm)	14.2 ± 9.5	11.3 ± 7.7	11.3 ± 2.3	-	35.0 ± 14.3
Wall thickness (µm)	5.2 ± 2.9	3.4 ± 2.3	5.4 ± 3.2	-	8.0 ± 1.6
Total width (µm)	20.3 ± 10.0	20.5 ± 13.8	19.4 ± 3.8	24.0	39.6 ± 9.8

Categorical data presented as n (%), and quantitative data shown as average ±standard deviation. There were no statistical differences between groups.normal

### 3.3 Lymphatic vessels in dura mater of temporal skull base

**FIGURE 3:**
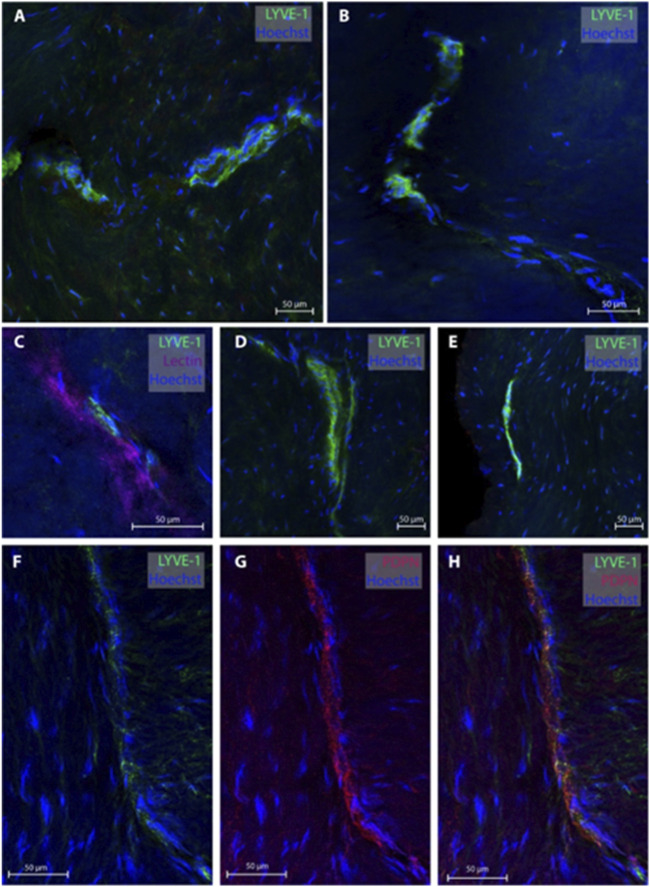
Lymphatic vessels in dura mater of temporal skull base. Representative images of lymphatic vessels in the temporal skull base region labeled with LYVE-1. Panels **(A, D, E)** are sections from patient #4, panel **(B)** from patient #3 and panel **(C)** from patient #2. In Panel C, the lymphatic vessels can be seen next to a lectin-labeled blood vessel. Panels **(F–H)** are a lymphatic blood vessel co-labeled with LYVE-1 and PDPN from patient #3.

Regarding the temporal skull base, dura samples from five individuals (patients #2, 3, 4, 10 and 11) were obtained, end 26 MLVs were examined, see representative images in Figure 3. In five subjects, the MLVS did not have blood vessels in proximity while the remaining three individuals presented with MLVs in proximity to a lectin-labeled blood vessel. These temporal MLVs had a lumen of 11.3 ± 2.3 µm, a wall thickness of 5.4 ± 3.2 µm and a total width of 19.4 ± 3.8 µm (Table 2).

### 3.4 Lymphatic vessels in dura mater of upper frontal convexity

**FIGURE 4:**
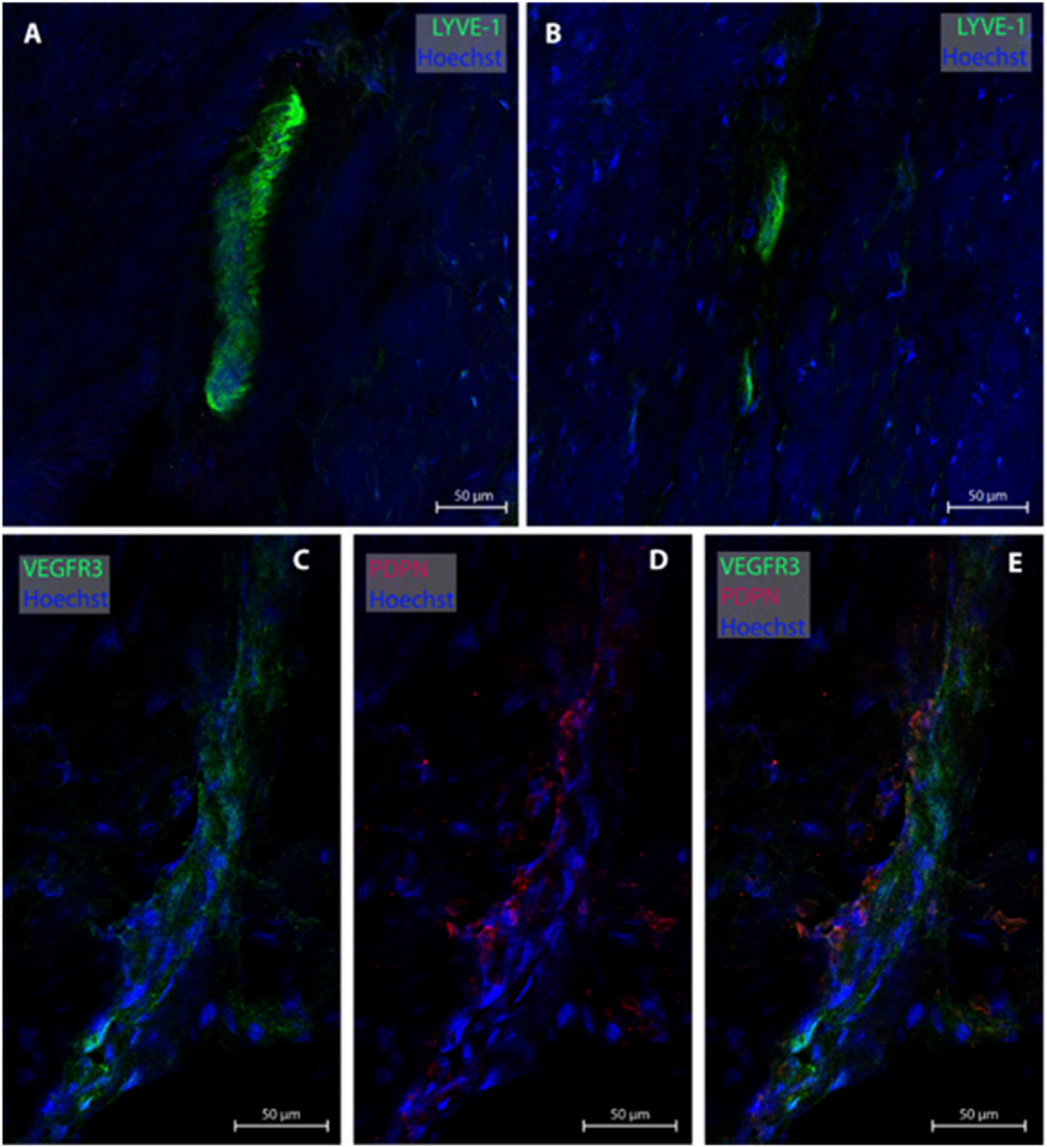
Lymphatic vessels in dura mater of upper frontal convexity. In the upper frontal convexity, two lymphatic vessels were found. One lymphatic vessel was labeled with LYVE-1 in consecutive sections **(A, B)**. The second vessels was co-labeled with VEGFR3 and PDPN **(C–E)**. All sections belong to patient #8.

One of the present study participants had a dura sample from the frontal convexity (patient #8). In this case, three MLVs were examined, all without any blood vessel in proximity. Representative images are shown in Figure 4. Total with was 24.0 µm (Table 2).

### 3.5 Lymphatic vessels in dura mater at cranio-cervical junction

**FIGURE 5:**
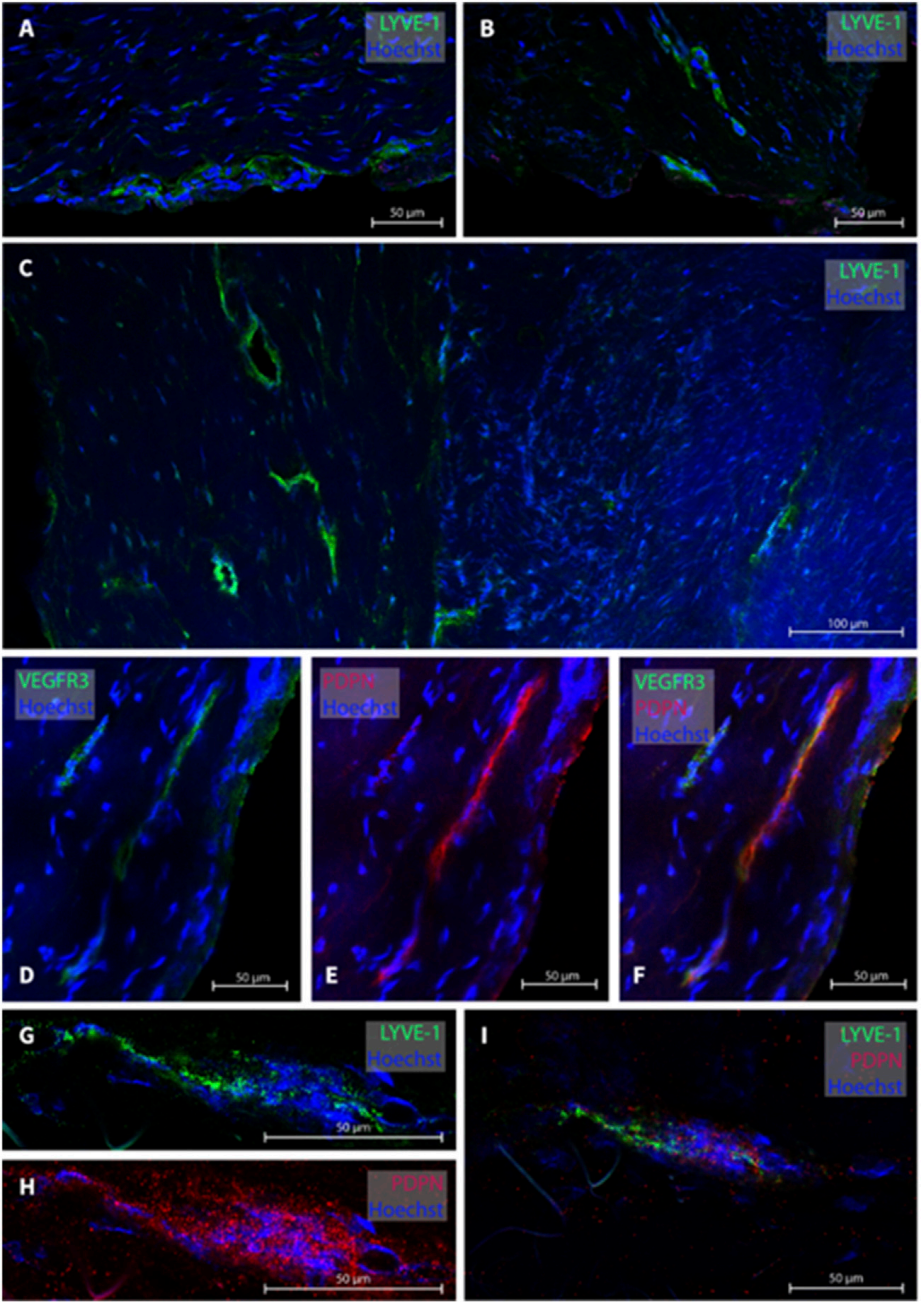
Lymphatic vessels in dura mater at cranio-cervical junction. In the cranio-cervical junction, several lymphatic vessel were labeled **(A–C)**. Panels **(D–F)** are a lymphatic vessel co-labeled with VEGF3R and PDPN while in panels **(G–I)**, LYVE-1 and PDPN was used. The sections belong to patient #15.

In the cranio-cervical junction (CCJ) the dura mater sample of one subject (patient #15) was analyzed. Several MLVs were found from which four were examined, see Figure 5 for representative images. The total width of MLVs here was 39.6 ± 9.8 µm, with a lumen of 35 ± 14.3 µm and a wall thickness of 8 ± 1.6 µm (Table 2).

### 3.6 Different characteristics of lymphatic vessels in different locations

Table 2 provides a comparative overview of the dimensions of lymphatic vessels found in the different dura locations. It might seem as the MLVs found at the posterior fossa and CCJ are slightly larger than the ones in both the temporal and frontal regions, but variability is too large to make firm conclusion.

## 4 Discussion

Our study demonstrates that in human dura mater, MLVs are present distant from sinus veins, and may be more widespread in dura mater than previously assumed. MLVs remote from blood vessels were rather common.

The presently used antibodies against the proteins LYVE-1, VEGFR3 and PDPN are well-established immunohistochemical markers of lymphatic endothelial cells (Louveau et al., 2015). The specificity of these markers was validated by co-labeling with LYVE-1, VEGFR3 and PDPN and by ruling out unspecific binding from the secondary antibody and auto-fluorescence. We also controlled the specificity by staining in mouse liver as positive control and mouse spleen as negative control (Supplementary Figure S1).

To the best of our knowledge, lymphatic vessels several centimeters away from sinus veins have not been shown immunohistochemically in humans before, except for in cadavers (Mezey et al., 2021). The present data suggests that lymphatic vessels could be found throughout the entire dura mater, both close to and remote from blood vessels. From this, we suggest that lymphatic vessels may have a more important role than if they were exclusively lining the dural veins, for example, regarding CSF clearance and immune neuromodulation (Rustenhoven and Kipnis, 2022). A growing body of evidence suggests that meningeal lymphatic vessels may be instrumental for disease states such as traumatic brain injury, neurodegenerative diseases (e.g., Alzheimer and Parkinson diseases), as recently reviewed (Bush et al., 2022). For example, recent experimental data suggest that MLVs have an important role in regulating the immune response against brain tumors (Hu et al., 2020; Song et al., 2020), traffic of T-cells during infections (Divan et al., 2018), and meningeal clearance of neurotoxic waste such as amyloid β to extra-cranial lymphatics (Pappolla et al., 2014; Da Mesquita et al., 2018; Da Mesquita et al., 2021), and clearance of blood from intracranial bleeds (Liu et al., 2020; C et al., 2020). In this regard, it is of interest that meningeal lymphangiogenesis induced by neuroinflammation seems heterogenous for the different central nervous system related locations (Hsu et al., 2019).

A significant role of widespread MLVs would be expected if substances were passing freely between the CSF spaces and the dura where the lymphatic vessels reside. Previously, tight junctions joining the cells of the outer arachnoid matter were thought to make the parietal layer of the arachnoid membrane impermeable to CSF (Weller, 2005; Weller et al., 2018). In this regard, it is of interest that “hot spots” were seen in mice along lymphatic vessels where these penetrate the arachnoid membrane (Louveau et al., 2018). Recent tracer studies in humans show passage of tracer from CSF to parasagittal dura (Ringstad and Eide, 2020), skull bone marrow (Ringstad and Eide, 2022) and enrichment of tracer in extra-cranial lymph nodes (Eide et al., 2018). Moreover, emerging human evidence suggests that solutes and cells passing from CSF to inside dura mater nearby the superior sagittal sinus have the potential to reach the lymphatic vessels since fluid flows easily into their lumen due to their non-traditional tight junctions (Baluk et al., 2007). Therefore, in humans, passage of substances from CSF to dura and MLVs occur. There may, however, be some differences compared with animals. While lymphatic egress via the cribriform plate to nasal mucosa seems to be important in rodents and other animal species (Johnston et al., 2004), this pathway seems to be less important in humans (Melin et al., 2020).

From the present observations, we speculate that larger caliber MLVs in humans are likely to be found close to the dural sinus veins and as the distance increases these vessels become smaller and less dense resembling “initial/capillary” lymphatics. By extension, the larger, denser, and more branched lymphatic vessels should be found as these vessels approach also the cervical lymphatics connecting with the extracranial lymphatic system. In other words, it is likely that the largest MLVs are basal and closer to the sinus veins while the smallest MLVs are distant to the sinus veins and in frontal and temporal regions of the dura. Further studies are need to confirm this.

The present data provide some indication that the MLVs to some degree differ between the various locations since MLVs located in the posterior fossa and the cranio-cervical junction were somewhat larger than the vessels found more dorsally (frontal convexity and temporal regions). This is also consistent with evidence from mice showing that basal MLVs have a bigger diameter and branch more densely as compared to dorsal MLVs (Ahn et al., 2019). However, firm conclusions cannot be made from the present observations as number is too low. We may neither conclude about the quantitative distribution of lymphatic vessels throughout the human dura. Cadaver studies might be preferable to answer this. We presently do not know the extent by which disease processes impact lymph angiogenesis and thereby the abundance of lymphatic cells in dura. It also should be commented that measurements of dimensions of lymphatic vessels regarding wall thickness, lumen and total width have methodological weaknesses. In future studies, stereology and 3D assessment of tissue specimens might be used to compare different dura locations and variation between diseases.

In conclusion, the presence of lymphatic vessels in human dura mater is not restricted to the vicinity of sinus veins, but are present in dura mater distant from the sinus veins. The functional implications of widely distributed MLVs need to be explored in future studies.

## Data Availability

The raw data supporting the conclusions of this article will be made available by the authors, without undue reservation.
