# Supplementary material for: Widespread distribution of lymphatic vessels in human dura mater remote from sinus veins
Source: Front Cell Dev Biol. 2023 Sep 19;11:1228344. doi: 10.3389/fcell.2023.1228344 (PMC10546208; doi:10.3389/fcell.2023.1228344)
Supplement: Supplementary file 1 [file DataSheet1.PDF]

Supplementary Material

Widespread distribution of lymphatic vessels in human dura mater remote from sinus veins

César Luis Vera Quesada, MD, MSc[1,2] Shreyas Balachandra Rao, MSc, PhD,[3] Reidun Torp, MSc, PhD[3] Per Kristian Eide, MD, PhD[1,2]

[1]*Department of Neurosurgery, Oslo University Hospital-Rikshospitalet, Oslo, Norway*
[2]*Institute of Clinical Medicine, Faculty of Medicine, University of Oslo, Oslo, Norway,*
[3]*Division of Anatomy, Department of Molecular Medicine, Institute of Basic Medical Sciences, University of Oslo, Oslo, Norway*

Corresponding author:
Professor Per Kristian Eide, MD PhD
Dept. of Neurosurgery,
Oslo University Hospital – Rikshospitalet,
PB 4950 Nydalen, 0424 OSLO, Norway
Phone: +47 91649419; Fax: +47-23074310
E-mail: p.k.eide@medisin.uio.no

Supplementary Figure 1

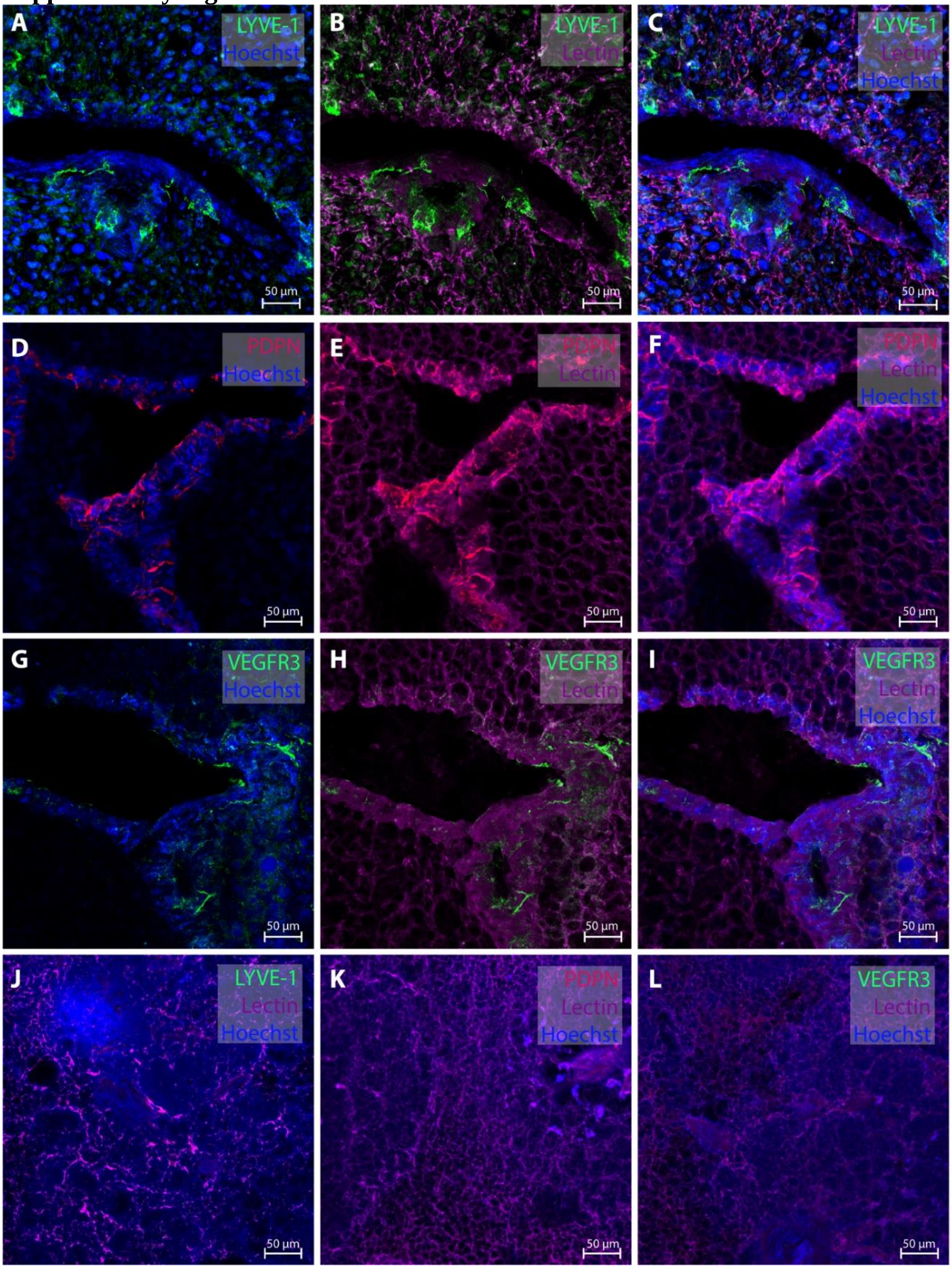

Positive and negative controls of antibodies in mouse tissue. Panels **A-I** show, as positive controls, roughly the same area of consecutive mouse liver sections with positive staining for LYVE-1 (**A-C**), PDPN (**D-F**) and VEGFR3 (**G-I**). Panels **J-L** show negative controls of mouse spleen sections where LYVE-1, PDPN or VEGFR3 labeling is absent as expected.

Supplementary Figure 2

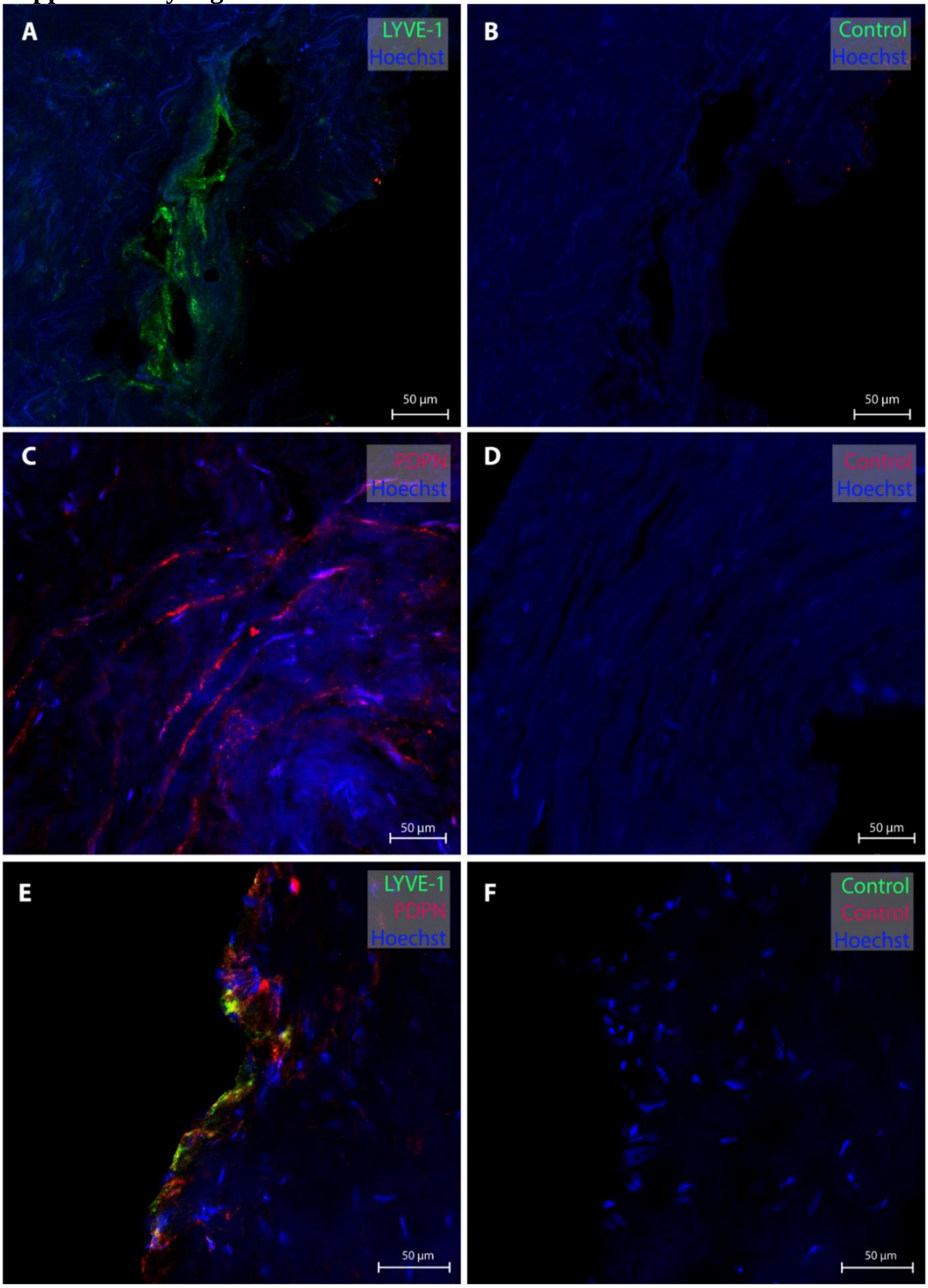

Control experiments showing specific antibody binding. The sections on the left column (**A**, **C**, **E**) show PDPN and LYVE-1 antibodies with CY2 and CY3 as secondary antibodies respectively. Sections on the right column (**B**, **D**, **F**) are consecutive to sections on the left used as controls where no primary antibodies were added. Panels **A** and **B** belong to patient #5, **C** and **D** to patient #12 and **E** and **F** to patient #15. Panels **C** and **E** in this figure are the same images previously shown in Figure 2. Lymphatic vessels in dura mater of posterior fossa panels **D** and **F**.
